# Characterisation of a New Family of Carboxyl Esterases with an OsmC Domain

**DOI:** 10.1371/journal.pone.0166128

**Published:** 2016-11-16

**Authors:** Mai-Britt V. Jensen, Louise E. Horsfall, Caroline Wardrope, Peter D. Togneri, Jon Marles-Wright, Susan J. Rosser

**Affiliations:** 1 Institute of Quantitative Biology, Biochemistry, and Biotechnology, School of Biological Sciences, University of Edinburgh, Edinburgh, United Kingdom; 2 Institute of Molecular, Cellular, and Systems Biology, College of Medical, Veterinary and Life Sciences, University of Glasgow, Glasgow, United Kingdom; 3 School of Biology, Newcastle University, Newcastle upon Tyne, United Kingdom; Danish Cancer Society Research Center, DENMARK

## Abstract

Proteins in the serine esterase family are widely distributed in bacterial phyla and display activity against a range of biologically produced and chemically synthesized esters. A serine esterase from the psychrophilic bacterium *Pseudoalteromonas arctica* with a C-terminal OsmC-like domain was recently characterized; here we report on the identification and characterization of further putative esterases with OsmC-like domains constituting a new esterase family that is found in a variety of bacterial species from different environmental niches. All of these proteins contained the Ser-Asp-His motif common to serine esterases and a highly conserved pentapeptide nucleophilic elbow motif. We produced these proteins heterologously in *Escherichia coli* and demonstrated their activity against a range of esterase substrates. Two of the esterases characterized have activity of over two orders of magnitude higher than other members of the family, and are active over a wide temperature range. We determined the crystal structure of the esterase domain of the protein from *Rhodothermus marinus* and show that it conforms to the classical α/β hydrolase fold with an extended ‘lid’ region, which occludes the active site of the protein in the crystal. The expansion of characterized members of the esterase family and demonstration of activity over a wide-range of temperatures could be of use in biotechnological applications such as the pharmaceutical, detergent, bioremediation and dairy industries.

## Introduction

Carboxylesterases (EC 3.1.1.1.) and lipases (EC 3.1.1.3) catalyse the hydrolysis and synthesis of ester bonds across a large variety of substrates. Esterases show a preference for water-soluble short chain fatty acids (<10 carbon atoms), while lipases prefer water-insoluble longer chain fatty acids (>10 carbon atoms) [1]. Esterases generally show promiscuous activity with a specificity for either the alcohol or acid moiety [1,2]. They are members of the α/β hydrolase superfamily with most using a catalytic triad of serine, aspartic acid and histidine in the active site, where the serine residue (found in the sequence motif GxSxG), is responsible for the nucleophilic attack on the substrate [1,3–5]. Esterases are important enzymes in several industrial processes [2,6], and extremophiles have the potential to produce enzymes with a broader tolerance to extremes of pH, temperature, salt, and activity in non-physiological solvents, where enzymes isolated from mesophiles are inactive. Cold active enzymes have come into focus in recent years due to their uses in the food and agricultural industries as well as bioremediation and low-energy waste water treatment in cold climates [7,8], as they can potentially achieve higher catalytic activity at low temperatures without the need for high-energy expenditure on heating reaction mixtures, and can also be readily inactivated with a temperature increase. The demand for new catalysts in biotechnology is ever increasing and most current commercial enzymes are derived from microbial sources. Due to the ease and low cost of sequencing microbial genomes, a wealth of information is now available for identification of putative new enzyme families with enhanced activity over currently available enzymes. An esterase EstO from the psychrotolerant bacterium *Pseudoalteromonas arctica* was previously characterised [9] with an N-terminal OsmC (osmotically induced family of proteins)-like domain. The removal of this domain resulted in higher activity; enhanced thermostability; and altered the tolerance of the enzyme to certain metal ions as well as EDTA. A bioinformatics search identified putative esterases related to EstO with the OsmC-like domain in a variety of bacterial species, including several extremophiles. To study this new family of serine esterases we characterised seven members of the family. An enzyme from the thermophilic bacterium, *Rhodothermus marinus* showed particularly high activity, even at low temperatures and was subjected to further kinetic characterisation and crystal structure determination. The results presented here, demonstrate features of an enzyme from a thermophilic organism that could be used in cold-adapted industrial processes.

## Materials and Methods

### Chemicals

4-nitrophenyl (4NP) esters, gum arabic, and imidazole were purchased from Sigma-Aldrich, Dorset, UK with the exception of 4-nitrophenyl benzoate was from Alfa Aesar, Lancashire, UK. and NaCl from VWR Leicestershire, UK, which also supplied Na_2_CO_3_. Carbenicillin, sugars, buffers and ethanol were purchased from Fisher Scientific, Leicestershire, UK. Tryptone and yeast extract were from ForMedium, Norfolk, UK. Primers were purchased from and sequencing was performed by Eurofins MWG Operon, Germany. Invitrogen, Paisley, UK supplied all vectors and *E*. *coli* strains used in this study.

### Identification of esterase sequences

A Blast search was performed on the amino acid sequence of EstO against the non-redundant protein sequence database with default parameters and sequence alignments were generated using ClustalΩ [10]. After manual inspection of the sequence alignments the C-terminal OsmC-like domain and linking region were removed from EstO sequence generating ΔEstO as in [9] and truncated variants of EstO homologues were generated based on the consensus domain boundaries obtained from inspection of these alignments.

### Bacterial strains and plasmids

*Pseudoalteromonas arctica* (DSM 18437), *Labrenzia aggregata* (DSM 13394), *Ensifer meliloti* (DSM 1981), *Roseobacter denitrificans* (DSM 7001), *Rhodothermus marinus* (DSM 4252), *Catenulispora acidophila* (DSM 44928), and *Cellulophaga algicola* (DSM 14237) were purchased from the DSMZ catalogue (Germany). All strains were grown in a multitron shaking incubator (Infors UK Ltd., Surrey) with shaking at 200 rpm in media and growth conditions detailed on the DSMZ website. Genomic DNA was isolated using the Gentra Puregene yeast/bact kit according to the manufacturer’s instructions (Qiagen Ltd, Sussex, UK). The esterase genes were amplified by PCR using KOD Hot Start DNA polymerase (Merck Chemicals Ltd., Nottingham, UK) and the oligonucleotide primers described in Table 1 to include the sequence CACC at the beginning of each gene. The genes were then cloned into pENTR^™^/D-TOPO^®^ vectors and sequenced before being inserted into Gateway^®^ pDEST^™^17 vectors to create the expression plasmids pDΔEstO, pDΔEstLA, pDΔEstEM, pDΔEstRM which encode the esterases with an N-terminal His_6_ tag. Alternatively, the genes were amplified by PCR using Platinum *taq* polymerase (Invitrogen, Paisley, UK) and primers were designed specifically to the start and end of the gene (no stop codon) (Table 1) for insertion into pEXP5-CT TOPO, to create the plasmids pEstRD-CT, pEstCA-CT, and pEstCAL-CT, which encode the truncated versions of the esterases with a C-terminal His_6_ tag. Site-directed mutagenesis of ΔEstRM and EstRM was performed using the QuickChange^®^ site-directed mutagenesis kit (Agilent, Cheshire, UK) according to the manufacturers instructions. All mutations were verified by sequencing.

**Table 1:** Primers used for gene amplification and site-directed mutagenesis.

Primer name	Primer sequence
EstO cacc for	caccatgcgacaaaaagtatcttttaaaagcg
deltaEstO rev	ttagtacttaacataacggtttgcccacg
EstLA cacc for	caccatgggacagcacccgctg
deltaEstLA rev	tcagtccttcacctcgtcgtcgg
EstCAL ns for	atgaaaaataaaacaatatcattcaagaactcaaaagg
deltaEstCAL ns rev	aatatatcttgatgcccattgtgcaattag
EstRD ns for	atgccaacagaacgaattgcctttgcc
deltaEstRD ns rev	gaaccccttcgggtccgc
EstCA ns for	atgtccacctcgctcaagg
deltaEstCA ns rev	ctcgggaagatagcggctg
EstEM cacc for	caccatggcattcaatacgcaacggc
deltaEstEM rev	tcacctcacgtggacatgttcgatcg
EstRM cacc for	caccatgcagatcaaaaccgttacgtttg
deltaEstRM rev	tcaacgacgtcccacgtaacgcg
fl EstRM rev	gtcgcgcaatcgggttcg
EstRM S109A F	tcggccacgctctgggtggagctgcagtgctggccgttgcg
EstRM S109A R	cggccagcactgcagctccacccagagcgtggccgatcagcag
EstRM S72A F	tttaccggcctaggagaagccgaaggagatttttccg
EstRM S72A R	ctccttcggcttctcctaggccggtaaaatcgaagcg

### Expression and purification of His_6_ tagged esterases

Plasmids were transformed into *E*. *coli* BL21-AI cells producing single colonies on LB agar plates supplemented with 50 μg/ml carbenicillin and 0.1% glucose. Recombinant protein expression used a single colony transformant to inoculate 100 ml LB with supplements as above. Cultures were incubated overnight at 37°C with orbital shaking at 200 rpm and next day the pre-culture was used to inoculate 2 l of LB media of the same composition. These were then grown at 37°C with orbital shaking at 200 rpm until an OD_600_ of 0.5–0.8 was reached. Recombinant protein expression was induced by adding 0.2% arabinose and cultures were incubated under the same conditions or moved to 28°C for 4 to 24 hours depending on the protein expressed. Cell pellets were collected by centrifugation at 7,000 x *g* for 30 mins at 4°C and flash-frozen in liquid nitrogen. The pellet was then resuspended in 10 x (v/w) buffer A (0.05 M Tris-HCl, pH 7.2, 0.5 M NaCl, 0.05 M imidazole) with 1 μl benzonase (Merck, Darmstadt, Germany) and disrupted using a French press. Cell debris was removed by centrifugation at 14,000 x *g* for 1 hour at 4°C and the supernatant was loaded onto a column previously charged with NiCl_2_ and equilibrated in buffer A. The column was then washed with buffer A, before an imidazole gradient of 0.05 M to 0.5 M in 5 column volumes was used to elute the enzyme. Fractions were analysed by SDS-PAGE and fractions containing the protein of interest corresponding to the estimated molecular weight were dialysed against 50 mM tris-HCl, pH 7.2 (supplemented with 0.3 M-0.5 M NaCl as necessary to ensure protein solubility) prior to filter sterilization using a 0.22 μm syringe filter, and were stored at 4°C. Buffer pHs were adjusted according to the calculated pIs of the proteins.

### Protein purification for crystallography and EstRM analysis

EstRM was purified as described above with further purification by size-exclusion gel filtration chromatography using a Superdex S200 16/60 column (GE Healthcare, Buckinghamshire, UK). The column was pre-equilibrated with 50mM Tris-HCl, pH 8.0, 150 mM NaCl and protein was loaded before running with 1.5 column volumes of buffer with the collection of 1.8 ml fractions. Fractions were analysed by 10–15% SDS PAGE and those containing the protein of interest were pooled and concentrated using a 10,000 Da MW cutoff centrifugal concentrator (Vivaspin, GE Healthcare, Buckinghamshire, UK)

### Measuring enzymatic activity

The concentration of purified enzyme was estimated using the protein absorbance at 280 nm and the extinction coefficient, calculated by the ProtParam tool available on the Expasy website [11]. Hydrolysis of 4-nitrophenyl benzoate was determined according to Winkler and Stuckmann and Al Khudary *et al*. with slight alteration [9,12]. A substrate emulsion was made by mixing 10 ml of ethanol containing 37 mg 4-nitrophenyl benzoate (15 mM) with 90 ml tris-HCl buffer (25 mM, pH 8.5) containing 100 mg gum arabic. 100 μl enzyme was then combined with 900 μl of substrate emulsion and the reaction incubated for 30 min at the required temperature, it was then stopped by placing on ice for 5 min followed by the addition of 100 μl of 25% Na_2_CO_3_. The reactions were centrifuged for 5 min at 13,000 rpm and 4°C, their absorbance was measured at 410 nm using a SpectraMax Plus384 spectrophotometer and SoftMax Pro programme (Molecular Devices, Berkshire, UK) against a control which contained buffer (25 mM Tris-HCl, pH 8.5.) rather than enzyme.

### Enzyme characterisation

To establish the optimum reaction conditions for the esterases, the esterase activity was measured at a pH range from 5.0 to 10.0 in universal buffer [13] with 1.5 mM 4-nitrophenyl benzoate as substrate with incubation at 30°C for 30 mins and, in another experiment, the esterase activity was measured at a temperature range 5–50°C in Tris-HCl buffer (25 mM, pH 8.5). The activity toward other 4-nitrophenyl esters was measured in the same manner using 1.5 mM of each substrate. The effect of metal ions on enzyme activity was measured by preincubating the enzyme with 10 mM metal chloride at 25°C for 2 hours, followed by the 30 min incubation assay at 30°C with 4-nitrophenyl benzoate substrate as previously described. Inactivation by (1 mM and 10 mM) EDTA, (0.4 mM and 4 mM) pefabloc, (0.1 mM and 1 mM) DTT and by 10% Tween20 was measured by pre-incubating the enzyme with the inactivator for 2 hours. The pre-incubation was then used with 4-nitrophenyl benzoate as substrate in the 30 min incubation assay, in Tris-HCl buffer (25 mM, ph 8.5) with 1 mg/ml gum arabic, as described earlier. As a control, the enzyme activity measured similarly but without the addition of any inactivator was defined as 100%.

### Enzyme kinetics

Enzyme activity assays of 4-nitrophenyl-esters were carried out in assay buffer (25 mM Tris.HCl pH 8.5, 5% Acetonitrile, 0.5% triton-100) and catalysis monitored by measurement of 4-nitrophenol production at 410 nm using a Spectramax plus 384 spectrophotometer (Molecular Devices) in real time (ε = 15,000 in assay buffer from standard curves). 10 mM stocks of 4-nitrophenyl esters were prepared by dissolving in 1 ml neat acetonitrile followed by addition of 9 ml assay buffer. Further dilutions were made using assay buffer. Saturation curves were fitted using SigmaPlot to obtain k_cat_ and k_M_ values for each substrate tested. Enzyme concentrations in reaction varied from 0.1–5 μM depending on the substrate tested, as there was considerable variability in rates of hydrolysis between the different enzymes.

### Effect of pH on activity

The effect of pH on the activity of full-length and truncated *R*. *marinus* esterase was performed in 25 mM universal buffer (25 mM boric acid, 25 mM phosphoric acid, 25 mM acetic acid; pH (5–10.5) adjusted to the required point by NaOH) containing 5% acetonitrile and 0.5% tritonX-100. Production of 4-nitrophenol from hydrolysis of 4-nitrophenyl-octanoate (1 mM) was monitored at 348 nm (Isosbestic point, ε = 4,147) over 5 minutes, and determining the absorbance at 410 nm per minute from the slopes generated. Concentrations of enzyme present were 0.57 μM and 0.74 μM for full-length and truncated respectively.

### Effect of metals and inhibitors

The effects of various metals and known enzyme inhibitors on esterase activity were studied by incubating enzymes with 1 mM and 10 mM DTT, EDTA, Pefabloc, PMSF or metal chloride for 1 hour before addition to substrate (1 mM 4-nitrophenyl-octanoate) in assay buffer (enzyme concentrations were the same as for pH assay) in 96-well plates. Catalytic activity was monitored by absorbance of 4-nitrophenol at 410 nm over 5 minutes as before, and determining the absorbance at 410 nm per minute from the slopes generated. Enzyme concentrations were 0.57 μM (full-length) and 1.0 μM (truncated).

### Temperature assay

To assay the effects of temperature on esterase hydrolysis of 4-nitrophenyl-octanoate, substrate and enzymes were pre-incubated at the desired temperature in a water bath for 5 minutes prior to mixing and further incubation for 5 minutes. The reaction was stopped by placing on ice, and absorbance at 410 nm was measured immediately using a quartz cuvette (samples containing substrate only were used as a blank in case of background hydrolysis). Enzyme concentrations were 0.1 μM (full-length) and 0.13 μM (truncated).

### Thermal shift assay

The fluorescence-based thermal shift assay [14] was used to determine the melting temperature (*T*_m_) of enzymes. It was performed in triplicate in total volumes of 50 μl (5 μM esterase in 25 mM Tris.HCl, pH 8, 1:1000 SYPRO^®^ orange (Sigma Aldrich) using an iCycler iQ^™^ (Bio-Rad, Herfordshire, UK) to monitor changes in fluorescence with increasing temperature (20–100°C in increments of 0.5°C, held for 30 seconds at each point).

### Peroxidase assay

The FOX assay [15] was performed in 25 mM potassium phosphate buffer (pH 7.4) plus 1 mM DTT using H_2_O_2_ and cumene hydroperoxide (both at 1 mM) as substrates. Horseradish peroxidase was used as a positive control, truncated esterase ΔCT (no OsmC domain) was expected to be negative for peroxidase activity. After addition of enzyme, aliquots were taken at five, 15 and 30 minutes, FOX reagent (Pierce) added and absorbance measured at 595 nm using a Spectramax plus 384 spectrophotometer (Molecular Devices). Concentration of substrates in solution was determined using extinction co-efficients (ε) obtained from standard curves.

### Statistical analysis

Results were analysed using Student’s *t*-test in SigmaPlot. Values of *p* < 0.05 were considered statistically significant. Significance was denoted as *, *p* < 0.05; **, *p* < 0.01.

### Protein crystallization

ΔEstRM protein at 15 mg/ml was crystallized by sitting drop vapor diffusion with 100 nl drops of protein supplemented with 100 nl of mother liquor comprising 0.2 M ammonium citrate dibasic, pH 5.0, 20% w/v PEG 3350 (Hampton PEG/Ion HT96 screen D12), drops were equilibrated against 70 μl of mother liquor for one month before crystals appeared.

### Structure determination and analysis

Crystals of ΔEstRM were mounted in 0.1 mm litholoops (Molecular Dimensions Limited) and cryoprotected by immersion in mother liquor supplemented with 20% (v/v) PEG 200. Crystals were flash cooled in liquid nitrogen and data was collected on I02 (25/07/2013) at Diamond Light Source. Diffraction data were integrated and scaled using XDS [16] and symmetry related reflections were merged with Aimless [17]. Data collection statistics are shown in Table 2. The resolution cut off used for structure determination and refinement was determined based on the CC_1/2_ criterion proposed by Karplus and Diederichs [18]. The structure of ΔEstRM was determined by molecular replacement using an ensemble of PDB entries: 2FUK [19], 3TRD, 3PF9 [20] as the search models. A single solution comprising a dimer in the asymmetric unit was found using Phaser [21]. The initial model was rebuilt using Phenix.autobuild [22] followed by cycles of refinement with Phenix.refine [23] and manual rebuilding in Coot [24]. The final model was refined with anisotropic B-factors for the protein chain and isotropic B-factors for ligands and water molecules. The model was validated using MolProbity [25]. Structural superimpositions were calculated using Coot. Crystallographic figures were generated with PyMOL (Schrodinger LLC). Data collection and refinement statistics are shown in Table 2. X-ray diffraction images are available online at the Edinburgh University Datashare repository (doi:10.7488/ds/1320).

**Table 2:** X-ray data collection and refinement statistics.

	EstRM
**Data collection**	
Wavelength (Å)	0.9795
Resolution range (Å)	44.62–1.56 (1.61–1.56)
Space group	P 1 2_1_ 1
Unit cell (Å)	*a* = 60.33, *b* = 74.07, *c* = 60.95
Unit cell (°)	β = 113.47
Total reflections	255,878 (25,615)
Unique reflections	69,888 (6,993)
Multiplicity	3.7 (3.7)
Completeness (%)	99.09 (98.48)
Mean I/sigma(I)	13.91 (1.88)
Wilson B-factor	23.37
R_merge_	0.044 (0.725)
R_meas_	0.051
CC_1/2_	0.999 (0.711)
CC*	1 (0.912)
**Diffraction images (DOI)**	10.7488/ds/1320.
**Refinement**	
Reflections used for R-free	3,544
R_work_	0.144 (0.264)
R_free_	0.176 (0.312)
Number of non-hydrogen atoms	3,914
macromolecules	3,614
ligands	20
water	280
Protein residues	466
RMS(bonds)	0.010
RMS(angles)	1.18
Ramachandran favored (%)	98
Ramachandran allowed (%)	1.78
Ramachandran outliers (%)	0.22
Clashscore	2.06
Average B-factor (Å^2^)	33.70
macromolecules	33.00
ligands	48.20
solvent	41.00
**PDB ID**	5CML

Statistics for the highest-resolution shell are shown in parentheses.

## Results

### Sequence analysis

**Fig 1:**
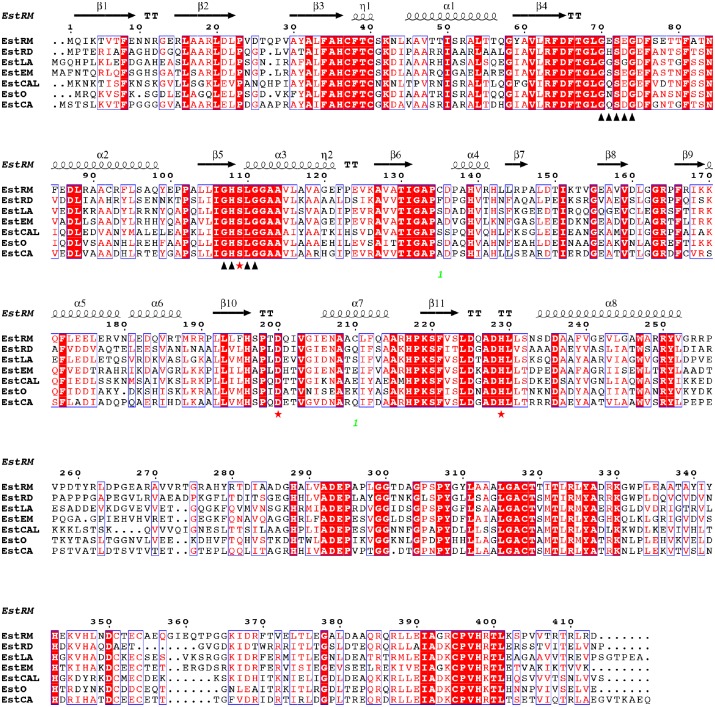
Multiple sequence alignment of OsmC esterase family proteins. Sequences of the seven OsmC esterase family proteins were aligned using ClustalΩ and displayed using ESPript [26]. Protein sequences are named as described in the results section. Secondary structure elements from the crystal structure of ΔEstRM are shown above the alignment. Strictly conserved residues are shown with a red background, while partially conserved residues are shown with red text. The two GxSxG motifs are identified with black triangles, while the conserved catalytic residues are shown with red stars. The esterase domain of the EstRM protein extends between the N-terminus and residue 255, with the OsmC domain from residue 256 to the C-terminus.

Previously, a paper by Al-Khudary *et al*., [9] identified a new type of serine esterase, EstO (UniProt ID: D6CHH1) from the psychrophilic bacterium *Pseudoalteromonas arctica*, composed of an N-terminal carboxyl esterase domain and a C-terminal OsmC-like domain. A BLAST search performed on the amino acid sequence for EstO, revealed putative uncharacterized proteins from several mesophiles, including the α-proteobacteria, *Roseobacter denitrificans* (RD, UniProt ID: Q166H3), *Ensifer meliloti* (EM, UniProt ID: Q92Y44); and *Labrenzia aggregata* (LA, UniProt ID: A0NLQ8). They are also present in flavobacteria, including *Cellulophaga algicola* (CAL, UniProt ID: E6X8P4). These proteins are also found in a number of extremophile bacteria, including the halophilic bacteroidetes *Salinobacter ruber*; the acidophilic actinobacteria *Catenulispora acidophila* (CA, UniProt ID: C7Q579); and the thermophilic: bacteriodetes *Rhodothermus marinus* (RM, UniProt ID: D0MHY8). Several of these were chosen for characterisation based on their protein sequence similarity and their different environmental niches, including both soil and marine bacteria. Although all of the EstO family members appear in multi-gene operons, they all show distinct genomic contexts with respect to the other genes in their loci. All of the putative enzymes identified contained the catalytic triad Ser, Asp, and His within their protein sequence and also contained a highly conserved nucleophilic elbow motif (GxSxG) (Fig 1) [1]. In addition, all the proteins contained a second, less well conserved, GxSxG pentapeptide found 32 residues upstream of the completely conserved pentapeptide (Fig 1). All proteins were of a similar size (44 to 47 KDa) and all had a C-terminal OsmC-like domain. A putative substrate-binding region containing two cysteine residues was identified towards the N-terminus of the protein (Fig 1), which is conserved in every family member identified so far.

### Expression in a heterologous host

The putative esterases were cloned into either pDest17 or pEXP5-CT TOPO (as described in materials and methods), which provided each protein with an N-terminal or C-terminal His_6_ tag fusion, respectively, for ease of purification using affinity chromatography. Truncated versions of the proteins were generated by removing the OsmC-like domains and are referred to below with Δ-prefix. Initial characterisation experiments were performed using the truncated esterases as these were expressed to a much higher degree in the heterologous system than the full-length versions and previous studies demonstrated higher esterase activities when the OsmC domain was removed [9]. For initial comparative characterisation, proteins were expressed in BL21-AI cells and purified using a nickel affinity column.

### Temperature and pH profiles

**Fig 2:**
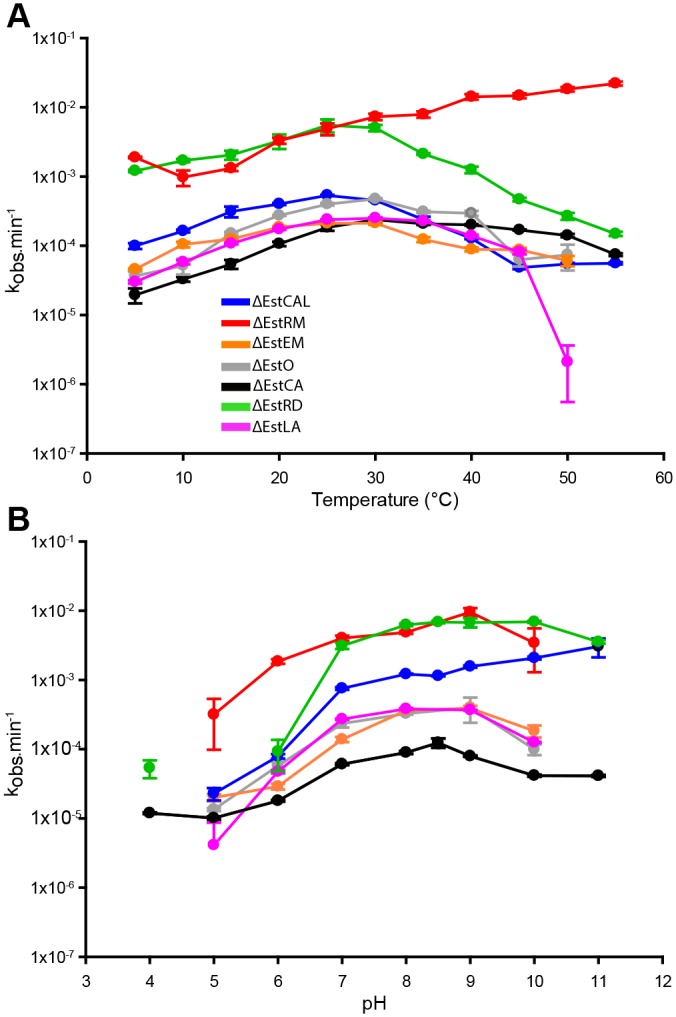
Characterisation of OsmC Esterase homologues. (A) Effects of temperature on OsmC esterase activities using 4-nitrophenol benzoate (1.5 mM) as substrate. (B) Effect of pH on OsmC esterase activities using 4-nitrophenol benzoate (1.5 mM) as substrate. The observed rate (k_obs_)_is defined as μM of 4-NP formed per μmole of enzyme. ΔEstCAL-Blue, ΔEstCA-black, ΔEstRD-green, ΔEstO-grey, ΔEstEM-orange, ΔEstRM-red, ΔEstLA-magenta. Results are presented as means ± S.D. of triplicate experiments.

Initial characterisation of the enzymes established optimum temperature and pH profiles of each enzyme using *p*-nitrophenyl benzoate as a substrate as described in [9]. The temperature profiles of the enzymes showed activity at every temperature tested between 5°C and 50°C (apart from ΔEstRM which was 5°C to 70°C). All but one enzyme showed an optimum temperature in the range 25–30°C (Fig 2A); the exception ΔEstRM, which is derived from a thermophile, had a higher optimum at 55°C. The influence of pH on enzyme activity was tested in the range 5.0 to 11.0 at 30°C. The optimum pH for ΔEstLA and the acidophile ΔEstCA was 8.0, while ΔEstRM, ΔEstEM, and ΔEstO had an optimum of 9.0. ΔEstRD had a slightly more alkaline optimum pH at 10.0, and ΔEstCAL showed the highest activity at pH 11.0 (Fig 2B).

### Effect of inhibitors

Enzymes were also preincubated for 2 hours with putative inhibitors to determine their sensitivity to these chemicals (S1A Fig). The serine hydrolase inhibitor Pefabloc reduced activity in all enzymes in a concentration dependent manner. However, a much less pronounced effect was seen with ΔEstEM, the activity of which was only decreased by 15% with 4 mM Pefabloc, while all other enzymes showed more than 70% reduction in activity. To investigate the need for divalent metal cofactors, the effects of the metal chelator EDTA were examined. At the lower concentration of EDTA (1.0 mM) no effects could be seen on the activity of ΔEstRM, while at 10 mM EDTA, it was decreased to 19% of the control. ΔEstEM was the most sensitive to EDTA with an 88% loss of activity at 1 mM, and no activity at 10 mM. An increase in activity was observed at 1 mM with ΔEstCA, however at 10 mM the activity decreased to less than 40%. ΔEstCAL, ΔEstLA and ΔEstRD lost activity at the lower concentration (90%, 70% and 30%, respectively), which was not exacerbated at the higher concentration. All the enzymes contain two conserved cysteine residues towards the N-terminal end of their sequence, so inhibitory effects through the disturbance of disulfide bonds of the reducing agent DTT were investigated, as disulfide bonds have previously been shown to be important for catalytic activity in some esterases [27,28] The inclusion of 0.1 mM and 1.0 mM DTT inhibited activity in all enzymes tested, although ΔEstEM and ΔEstCA were less affected at the higher concentration of DTT.

### Effect of metal ions

Further investigation of the proteins revealed that mono- and divalent- cations had different effects on the catalytic activity of EstO family members. The activity of the esterases on *p*-np benzoate was tested after pre-incubation with 10 mM of different divalent and monovalent metal chlorides (S1B Fig). ΔEstRM showed increased esterase activity with all metals tested apart from Fe^3+^ and Zn^2+^, which decreased activity. ΔEstLA showed increased activity with Mg^2+^ and K^+^, while all other metals had inhibitory effects. Little or no effect could be seen on ΔEstCAL activity with K^+^, Mg^2+^, and Co^2+^, and Ca^2+^, while all other metals decreased activity to less than 10% of the original activity. An increase in ΔEstEM activity was seen when incubated with Mg^2+^ and Ca^2+^, while it was unaffected by Fe^2+^. ΔEstEM was the only enzyme not affected by incubation with Zn^2+^. Remarkably, this enzyme was also the only one to show a decrease in activity when incubated with K^+^. Incubation with Ni^2+^ and Co^2+^ also resulted in loss of activity in this enzyme.

### Substrate preferences

The esterases were also tested against different *p*-nitrophenyl esters with varying chain length (C8-C18) and compared to the aromatic ester substrate used in previous experiments, to determine whether any of them showed unusual substrate preference profiles as esterases normally prefer shorter chain esters of less than 10 carbons [1]. The majority of the enzymes preferred the shortest chain ester tested (C8), while activities against carbon chains of over 12 atoms were less than 20% of the maximum (S2 Fig). It is worth noting that ΔEstEM, ΔEstRD, and ΔEstO showed a preference for decanoate (C10) and the latter also for dodecanoate (C12).

### EstRM kinetics

Given the very high activity of the esterase from the thermophile, full length and truncated versions of EstRM were investigated further and their kinetic parameters were established using different 4-NP ester substrates (Table 3). The truncated version had the highest rate with butyrate, but the highest affinities for benzoate and octanoate. The kinetic parameters for the full-length protein were very similar to those of the truncated version, however a decreased reaction rate with octanoate was observed. The fact that the esterase activity was improved when the OsmC-like domain was removed, suggests that the OsmC domain may affect the accessibility of the active site. The entire family of esterases described earlier, have two GxSxG pentapeptide motifs. Site directed mutagenesis was employed to change the Ser residue in either putative catalytic triad into alanine (S72A and S109A) to define which serine is required for activity. The S72A had the same *K*_m_ for butyrate as the WT protein, but a 6.7-fold decrease in turnover. With octanoate as a substrate both the *K*_m_ and reaction velocity were decreased by 1.3 and 10-fold, respectively. Mutagenesis of the S109 residue virtually abolished activity, identifying this as the most probable active site serine.

**Table 3:** Kinetic parameters for EstRM and ΔEstRM.

Substrate	kcat (s^-1^)^a^	K_m_ (mM)^a^	kcat/K_m_ (mM^-1^ s^-1^)
**ΔEstRM**			
4NP-acetate (4NP C2)	0.73±0.14	3.54±0.8	206
4NP-butyrate (4NP C4)	8.7±0.75	1.07±0.17	8131
4NP-octanoate (4NP C8)	0.21±0.0033	0.49±0.048	429
4NP-decanoate (4NP C10)	0.026±0.0013	0.825±0.13	32
4NP-dodecanoate (4NP C12)	0.009±0.0007	0.981±0.15	9
4NP-benzoate (4NP aromatic)	0.08±0.001	0.244± 0.012	328
**EstRM**			
4NP-butyrate (4NP C4)	7.76±0.46	0.962±0.14	8067
4NP-octanoate (4NP C8)	0.07±0.0031	0.309±0.07	227
4NP-benzoate (4NP aromatic)	0.088±0.0035	0.292±0.031	301
**ΔEstRM S72A**			
4NP-butyrate (4NP C4)	1.3±0.11	1.05±0.21	1238
4NP-octanoate (4NP C8)	0.02±0.0009	0.66±0.06	30

^a^ Average values (mean ± S.D.) from triplicate experiments

### Differences in activity between full length and truncated EstRM

**Fig 3:**
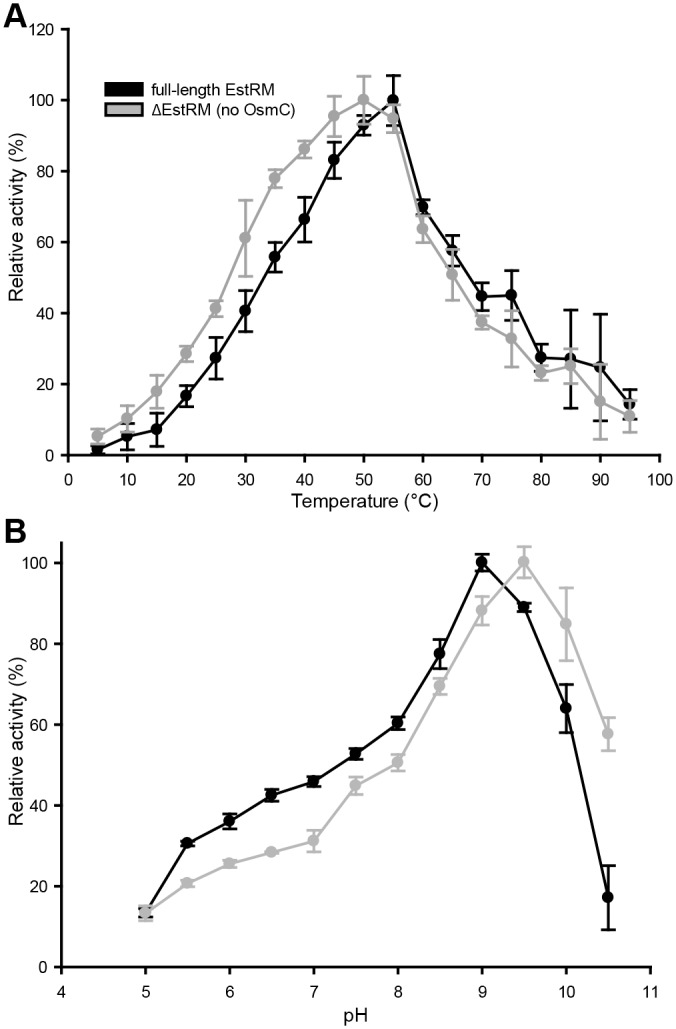
Effect of temperature and pH on full length and truncated EstRM. (A) Relative activity of ester hydrolysis at different temperatures. (B) Relative activity of ester hydrolysis at different pHs. Full-length EstRM is shown in black, the truncated esterase (ΔEstRM) in grey. 4-nitrophenyl-octanoate (1 mM) was used as a substrate to monitor ester hydrolysis. Relative activities expressed as percentages of maximal activity were plotted for each temperature and pH point. Results are presented as means ± S.D. of triplicate experiments.

**Fig 4:**
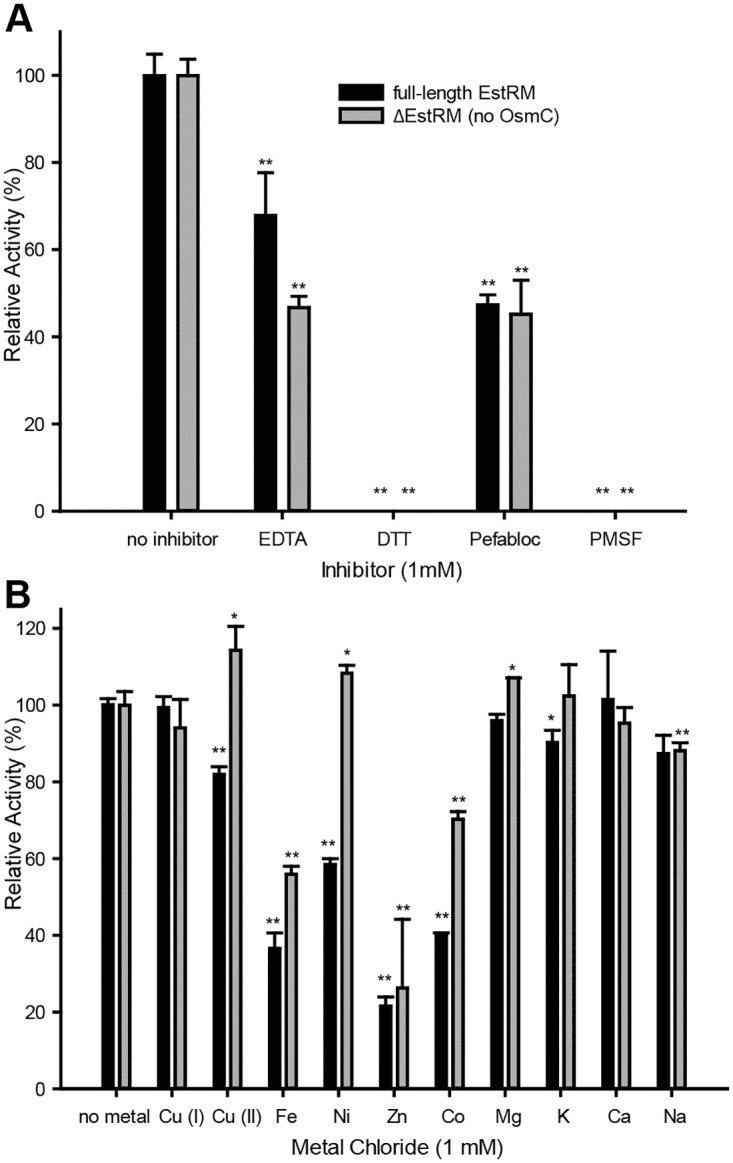
Relative activity of ester hydrolysis after incubation with known inhibitors and metal salts on activity of full length and truncated EstRM. (A) Enzymes were pre-incubated with 1 mM of EDTA, DTT, Pefabloc or PMSF for 1 hour before addition to substrate in assay buffer. (B) Enzymes were pre-incubated with 1 mM of CuI, CuII, Fe, Ni, Zn, Co, Mg, K, Ca, or Na chloride for 1 hour before addition to substrate in assay buffer. Substrate used to monitor activity was 4-nitrophenyl-octanoate (1 mM). Full-length EstRM is shown in black and truncated enzyme (ΔEstRM) in grey. Results were plotted as percentages of activity relative to measured activity when no inhibitor or metal salts were present. Results are presented as means ± S.D. of triplicate experiments.

The temperature optimum was shifted from 55°C for the full-length enzyme to 50°C for the ΔEstRM (Fig 3A). Thermal denaturation assays [14] showed a single peak at around 87°C for ΔEstRM, while a second peak around 70°C was seen for the full length EstRM (S3 Fig). When investigating pH optima the truncated version was found to be slightly more alkaliphilic with an optimum of 9.5 compared to 9.0 for the full-length (Fig 3B). All inhibitors affected both versions of the enzyme in similar ways (Figs 4A and S4A); the addition of either 1 mM or 10 mM PMSF resulted in complete loss of activity, while 1 mM Pefabloc only reduced the activity by 50%. The disulphide bond reducing agent DTT also abolished activity of both proteins at either concentration, suggesting that the two cysteines towards the N-terminus of the protein influence the catalytic activity of the protein, as the only other cysteines within the protein are located in the OsmC domain. Of the metals tested (Figs 4B and S4B), pre-incubation with Fe^3+^, Co^2+^, and Zn^2+^ resulted in a significant decrease in activity of both full length or truncated EstRM, although the full-length enzyme was significantly more sensitive to Co^2+^ and Ni^2+^ than the truncated enzyme, with both 1 mM and 10 mM metal salts present. Inclusion of 1 mM Cu^2+^ had opposite effects on the activities of the enzymes; full length was inhibited, while the activity of the truncated enzyme was slightly increased. However, this effect was not observed with 10 mM Cu^2+^, as the activity of both enzymes was reduced.

### OsmC domain

The OsmC domain found in this family of proteins is of particular interest, as it has not been seen linked in this way to another functional enzymatic domain such as an esterase before. The *E*. *coli* OsmC protein is able to metabolise both organic and inorganic peroxides [29]. While the OsmC domain found in the OsmC-esterase family shares only around 15% sequence identity to the *E*. *coli* OsmC protein, these proteins share the two conserved cysteine residues that form the active site of the *E*. *coli* peroxidase (cys59 and cys125 in the *E*. *coli* sequence). A number of the proteins in this study also possess an additional CXXC motif in the OsmC domain; this sequence is characteristic of many redox-active proteins [30–32]. To find out if the OsmC domain of full-length *R*. *marinus* esterase exhibits peroxidase activity, the ferrous oxidation xylenol orange (FOX) assay was performed to detect changes in peroxide substrate concentration upon addition of the full length enzyme. No significant peroxidase activity was detected when either inorganic H_2_O_2_ or cumene hydroperoxide (organic peroxide) were used as substrates, despite the presence of the conserved cysteines. An attempt was made to determine the three-dimensional structure of the full length EstRM to understand the relationship between the esterase and OsmC domains. While a single crystal of this protein was produced using commercial screens, we were unable to determine its structure in the absence of a good molecular replacement search model. Attempts to reproduce the crystallization with selenomethionine derivatized protein for experimental phasing were unsuccessful.

### Three-dimensional structure of ΔEstRM

**Fig 5:**
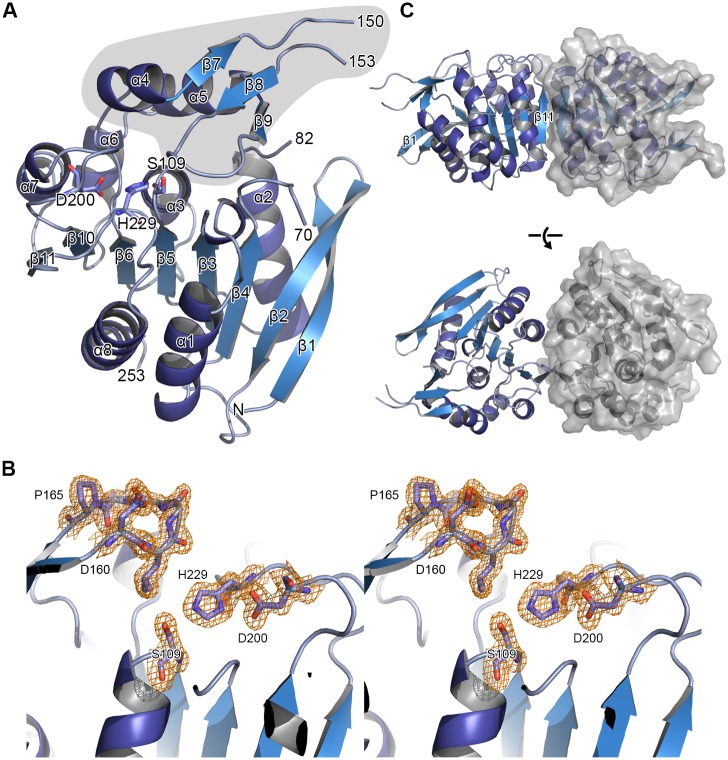
Crystal structure of ΔEstRM. (A) Cartoon view of ΔEstRM with secondary structure elements labelled and putative catalytic residues shown as sticks. The lid region that occludes the active site is highlighted in grey. (B) Wall-eyed stereo view of the lid region with representative 2mFo-DFc electron density map shown as an orange mesh and contoured at 1.5 σ. (C) Dimer of ΔEstRM present in the crystal structure. A cartoon view is shown for each chain, with the solvent accessible surface of one chain shown in grey.

While the full length OsmC esterases were recalcitrant to our attempts at structure determination, the putative esterase from *Rhodothermus marinus*, EstRM, was crystallised as a C-terminal truncation comprising residues 1–255 of the 410 amino acid full-length protein, including the alpha-beta-hydrolase domain, with a C-terminal hexa-histidine tag. The structure was determined by molecular replacement to 1.56 Å resolution. The structure was refined with anisotropic B-factors and the final model has an R_work_ of 0.144 and R_free_ of 0.176 (Statistics for the X-ray data collection and refinement are shown in Table 2). The structure consists of two molecules in the asymmetric unit, which align with an rmsd of 0.35 Å in their Cα positions over 225 residues and can thus be considered identical in their overall fold. Due to differences in the crystal-packing environment, the two molecules display some differences in surface exposed loops. Molecule A has visible electron density for residues 1–70, 82–150, and 153–253; while molecule B has visible electron density for residues 2–70, 84–148, 156–168, 175–253. ΔEstRM has a classical α/β-hydrolase fold [5] (Fig 5), with a core β-sheet made up of seven parallel strands (β1, β3–6, β10–11) and one antiparallel strand (β2); one face of the protein is flanked by two alpha helices (α1 and 8) and the other by four helices (α2, 3, 6, 7). There is an insertion in the α/β hydrolase fold between β6 and α6, which is formed by α4, β7, β8, β9, and α5, encompassing residues 136–180. Insertions in the α/β hydrolase fold are common and may modulate substrate accessibility to the active site of the protein [33]. The loop between β7 and β8 is not visible in the electron density for either chain, while β8 and β9 form a tight β-hairpin structure that occludes the proposed active site of the enzyme (Fig 5B). The structure of ΔEstRM has a partially occupied intra-molecular disulphide bond between residues C135 and C210 (S5 Fig). This disulphide links α7 with the loop preceding α4, stabilising the position of the beginning of the insertion domain. These cysteine residues are not conserved among other α/β hydrolase family proteins and appear to be unique to the ΔEstRM protein. The presence of the disulphide may enhance the thermostability of this enzyme in comparison to other members of the family, as the introduction of such features is common in thermotolerant enzymes [34,35]. The effect of the reducing agent DTT on the activity of this enzyme may be a consequence of the destabilisation of this disulphide bond and resulting effects on the active site-cleft of the protein caused by increased flexibility in the lid-region of the protein. The two molecules in the asymmetric unit are related by a two-fold rotation around an axis perpendicular to β10, β11, and α7 (Fig 5C). This dimer is unlikely to be physiological due to the fact that the protein elutes primarily as monomer on S200 size-exclusion chromatography based on calibration standards (S6 Fig). The results of PISA analysis [36] of the dimer interface indicate that it buries only 12% of the total area of the complex, and while this is stabilised by 8 hydrogen bonds and 5 salt bridges, this is considered to be an unstable interface on its own. The residues involved in forming these bonds (F221, S220/223 for hydrogen bonds; and R249/250, D236 in salt bridges) are not well conserved between the different OsmC-esterase family proteins. The hydrophobic residues involved in the dimer interface (residues 221–224) and other residues in β11 are well conserved; however, the side-chains of these residues mainly interact with residues in α7 and α8 and face towards the core of the monomer. The full-length protein also elutes from an S200 size-exclusion column primarily as a monomeric species, with a smaller peak corresponding to higher-order aggregates and possibly dimers (S6 Fig). Taken together these data suggest that the protein exists as a monomer in solution. Given the fact that α8 is at the C-terminus of the construct, it may be the case that the OsmC domain interacts with this region of the protein. In the absence of a structure of the full-length version of the *R*. *marinus* esterase we are unable to make any conclusions as to the position of this domain.

### Hydrolase active site and lid domain

**Fig 6:**
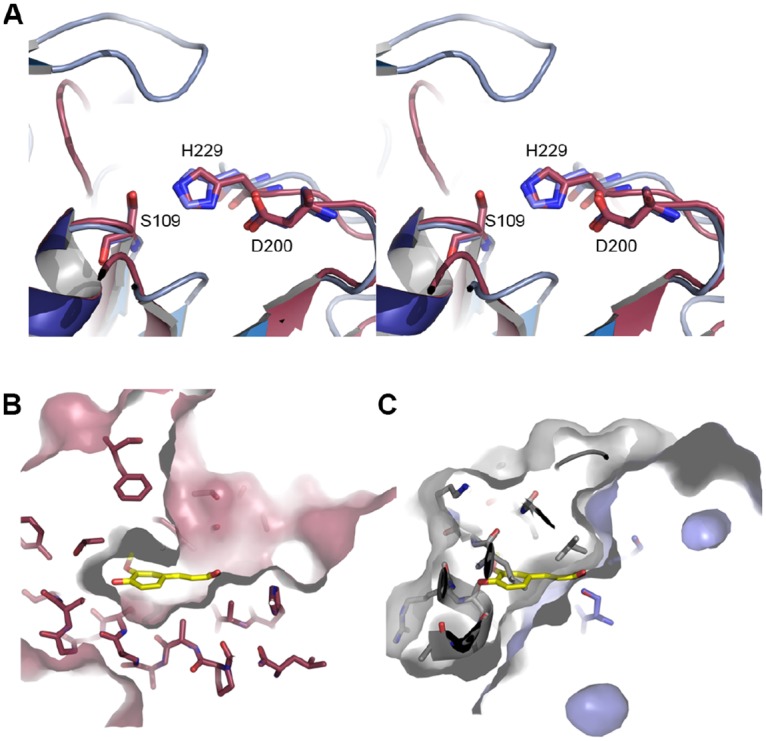
Active site of ΔEstRM. (A) Alignment of ΔEstRM with *Lactobacillus johnsonii* cinnamoyl esterase (PDBID: 3PFC)[20] displayed as a wall-eyed stereo view. ΔEstRM is shown in blue, while 3PFC is shown in raspberry. Active site residues are shown as sticks. The lid region of ΔEstRM is clearly visible at the top of the view and extends much further across the active site than the 3PFC lid. (B) Surface view of the active site of 3PFC, the ferulic acid ligand (shown as yellow sticks) is buried within a cleft between the main domain of the protein and the lid region. (C) ΔEstRM shown in the same view as (B) with an overlay of the ferulic acid; it is clear from this view that the active site is completely occluded by the lid-region, shown as a grey surface.

ΔEstRM possesses the classical α/β-hydrolase catalytic triad of a catalytic nucleophile (S109), acidic residue (D200) and Histidine (H229) [3]; mutagenesis of S109 abolished all of the esterase activity of this enzyme, confirming the identification of this residue as the active-site nucleophile. The inserted domain between β6 and α6 forms a lid that completely occludes the active site, with a loop formed by residues 160–165 sitting directly above S109 (Fig 6A). In this loop L161 is in apposition to S109 and H229. The loop shows excellent electron density and the residues in this loop in chain A have an average B-factor of 38 Å^2^ compared to 34 Å^2^ for the whole chain. The lid domain is connected to the main α/β-hydrolase domain through two well-structured loops, but has an overall higher average B-factor, 44 Å^2^, than the rest of the protein chain. This is primarily due to the regions leading to and from the disordered loop between residues 150–153, which show particularly high B-factors. The differences seen between the two chains in the asymmetric unit and high B-factors indicate that the lid region is likely to be flexible and may act to modulate the accessibility of the active site. The second GxSxG site around Serine 72 is part of a loop, between residues 70 and 82, that is disordered in the crystal structure and as such it is not possible to determine whether this is in close apposition to any Asp, or His residues to form a second active site.

### Structural comparison of EstRM *Lactobacillus johnsonii* cinnamoyl esterase

A comparison of ΔEstRM with the structure of the *Lactobacillus johnsonii* cinnamoyl esterase (PDBID: 3PFC) [20] show that the two proteins have an rmsd Cα deviation of 2.2 Å over 196 aligned residues for both the apo- and ligand-bound forms of the latter protein, in spite of the fact that the proteins share only 19% sequence identity [20]. In the structure of the *L*. *johnsonii* esterase, residues in the lid domain contribute to substrate discrimination and binding (Fig 6B). The lid region of ΔEstRM sits directly above the ligand binding-site of the *L*. *johnsonii* esterase (Fig 6B and 6C) blocking the active site and leaving it inaccessible to ligands in the crystal. The lid residues in contact with the active site are primarily hydrophobic, which may imply that this site is selective for acyl chains and unsubstituted aromatic groups. While the lid shares some of the hydrophobic character of the lids found in members of the lipase family, such as the human monoglyceride lipase [37], it adopts a distinct secondary structure and conformation above the active site. Attempts to crystallise the full-length EstRM protein and the ΔEstRM in complex with ligands and inhibitors proved to be unsuccessful and we were unable to determine the position of the lid domain with ligand bound, or the structure of the C-terminal OsmC domain. The catalytic function of the OsmC domain and its role within the context of the esterase remain to be seen, and warrant future investigation.

## Discussion

We have described seven members of a new family of serine esterases all containing a C-terminal OsmC-like domain taken from extremophilic bacteria from a wide range of ecological niches. The enzymes characterised displayed different sensitivity to metal ions and common serine esterase inhibitors. Notably, the ΔEstEM was partially insensitive to inhibition by Pefabloc, which could indicate enhanced substrate selectivity at the active site, protecting this enzyme against inhibitor compounds. The majority of members of this family of esterases contain multiple cysteine residues, capable of forming disulphide bridges. In the case of the ΔEstRM a single disulphide bridge is seen some distance away from the putative active site. These cysteine residues are not conserved in other members of the family and may specifically enhance the stability of this enzyme as is the case for many thermophiles [35]. The inhibitory effect of DTT on all enzymes tested indicates that one of these cysteines may be necessary for the activity of the enzyme, or a disulphide bridge may be required for enzyme stability. The established mechanism for carboxylesterase enzymes does not directly indicate the involvement of metal ions in catalysis [38]; however, the modest inhibitory effect encountered with EDTA suggests that certain metals could have a stabilising effect on the structure of the enzymes. An exhaustive study of the effects of metals on enzyme activity and the reversibility of these effects was beyond the scope of this study. The activity of the ΔEstRM and ΔEstRD enzymes over a broad temperature range was striking, of particular note was their activity against 4-np benzoate which was almost two orders of magnitude higher than other members of this family and significantly more active at cold-temperatures when compared to EstO, which is from a psychrophile. This particular attribute of the EstRM and EstRD proteins would be very attractive in industrial catalysis reactions where heat-labile substrates are present.

The crystal structure of ΔEstRM showed that this family conforms to the classical α/β-hydrolase fold with the insertion of a lid-domain that appears to mediate access to the active site cleft. Of note is the fact that removal of this lid in the *Candida antarctica* lipase A effectively negates the interfacial activation mechanism adopted by these enzymes [39]. While this mechanism displays a two-step response curve, the OsmC family esterases show classical Michaelis-Menten kinetics against the substrates tested in this study. Putative substrate binding residues were identified in the crystal structure of ΔEstRM and this region is conserved across all family members. Further study of the substrate binding pocket and lid-region seen in the ΔEstRM structure will allow a greater understanding of substrate selectivity and the influence this region has on the catalytic activity and kinetics of these proteins. The intra-molecular disulphide bond near the active site in ΔEstRM may contribute to its thermostability and it will be interesting to probe the influence of this on substrate discrimination and activity.

While it has previously been shown that OsmC proteins have peroxidase activity [29,40,41], the OsmC-like domains in this family did not contribute to the esterase activity of the full-length proteins, nor was any peroxidase activity detected against H_2_O_2_ or the organic cumene hydroperoxide. The function of the OsmC domain common to this family and its influence on the activity of these enzymes remains enigmatic. Characterisation of the activity of these esterases in their full-length and truncated forms showed that the OsmC domain reduced the activity of these enzymes against the longer chain substrates used. We have shown that simply removing the OsmC-like domain increases their esterase activity against short-chain substrates, with little effect on their stability. The fact that the OsmC domain does not show activity in model assay conditions, in spite of possessing the conserved catalytic cysteine residues common to this fold, does not rule out its enzymatic function. We were unable to determine the structure of the full-length ΔEstRM protein, so have no structural detail on the spatial relationships between the OsmC domain and the esterase domain. The presence of this domain in all members of this family implies a specific function that remains to be identified. It is possible that the fusion of these domains allows substrate channelling between the two enzymatic functions, perhaps allowing the capture of organic hydroperoxide-compounds and their subsequent breakdown and recycling by the esterase.

Cold active enzymes are attractive for use in industrial biotechnology applications, particularly as additives in biological cleaning products, textile, food and fragrances industries, and bioremediation [42]. These are commonly identified and isolated from psychrophilic organisms, or from the metagenomes of environmental isolates from arctic regions [43]. With such enzymes there is often an observed trade off between their stability and activity at cold temperatures [44,45]. In this work we have identified two members of a new family of serine esterases from thermophilic bacteria that display activity over a wide temperature range and display activity levels that are up to two orders of magnitude higher than other members of this family. Although we have not identified the natural substrates for these enzymes, the substrates used are related to both natural and synthetic esters with relevance to biotechnological applications. The increased stability and activity of our thermophilic enzymes make them suitable for harsh industrial processes and uses in the dairy, pharmaceutical, cosmetic, and detergent industries. Their substrate specificity and enantioselectivity could be altered through site directed mutagenesis or directed evolution towards longer or more complex carbon chain esters [46] and we have shown that simply removing the OsmC-like domain increases their activity, with little effect on their stability. Using a thermophilic enzyme for cold active applications gives enhanced enzyme stability with the potential for making changes to the active site to broaden the substrate range of the enzyme. Thus, the *Rhodothermus marinus* esterase is an excellent candidate for future exploration for industrial uses.

## Supporting Information

S1 Fig Relative activity of ester hydrolysis after incubation with known inhibitors and metal salts on activity of truncated OsmC esterases. (A) Effects of inhibitors on OsmC esterase activities using 4-nitrophenol benzoate (1.5 mM) as substrate. (B) Effects of metal salts on OsmC esterase activities using 4-nitrophenol benzoate (1.5 mM) as substrate. ΔEstCAL-Blue, ΔEstCA-black, ΔEstRD-green, ΔEstO-grey, ΔEstEM-orange, ΔEstRM-red, ΔEstLA-magenta. Results were plotted as percentages of activity relative to measured activity when no inhibitor or metals salts were present. Results are presented as means ± S.D. of triplicate experiments.
(PDF)

S2 Fig Relative substrate specificities of OsmC esterases. Enzyme assays were performed with 4-NP esters of varying chain length (C8-C18) and an aromatic ester (benzoate) as substrates (1.5 mM). ΔEstCAL-Blue, ΔEstCA-black, ΔEstRD-green, ΔEstO-grey, ΔEstEM-orange, ΔEstRM-red, ΔEstLAmagenta. Results were plotted as percentages of activity relative to substrate with highest measured activity of individual esterases. Results are presented as means ± S.D. of triplicate experiments.
(PDF)

S3 Fig Thermal stability of full length and ΔEstRM proteins. Thermal melting profiles for (A) full length and (B) ΔEstRM proteins. Unfolding of esterases was monitored between 20 and 100°C using SYPRO Orange fluorescent dye. The gradients of esterase unfolding were plotted as a function of temperature. Results are presented as means of triplicate experiments.
(PDF)

S4 Fig Relative activity of ester hydrolysis after incubation with 10 mM known inhibitors and metal salts on activity of full length and truncated EstRM. (A) Enzymes were pre-incubated with 10 mM of EDTA, DTT, Pefabloc or PMSF for 1 hour before addition to substrate in assay buffer. (B) Enzymes were pre-incubated with 10 mM of CuI, CuII, Fe, Ni, Zn, Co, Mg, K, Ca, or Na for 1 hour before addition to substrate in assay buffer. Substrate used to monitor activity was 4-nitrophenyl-octanoate (1 mM). Full-length EstRM enzyme is shown in black and truncated EstRM (ΔEstRM) in grey. Results are presented as means ± S.D. of triplicate experiments.
(PDF)

S5 Fig Wall-eyed stereo view of the intramolecular disulphide present in the ΔEstRM crystal structure. The protein backbone is shown as a cartoon view with the cysteine residues shown as sticks. The 2mFo-DFc electron density for this region is shown as an orange mesh at 1.5 σ. It is clear from the electron density that this disulphide is only partially occupied in this structure as multiple conformations of the sulphur atom are visible.
(PDF)

S6 Fig S200 size-exclusion gel-filtration chromatography of EstRM and ΔEstRM. Relative absorbance at 280nm is plotted against elution volume for both the full length EstRM and the ΔEstRM truncation. The major peaks at 76 ml (EstRM) and 82 ml (ΔEstRM) are consistent with the monomer size, while the minor peaks at 68 (EstRM) and 72 ml (ΔEstRM) represent a minor polulation of dimeric protein. The full-length EstRM trace shows a proportion of the protein aggregating into higher-order oligomers.
(PDF)
