# Supplementary material for: Characterisation of a New Family of Carboxyl Esterases with an OsmC Domain
Source: PLoS One. 2016 Nov 16;11(11):e0166128. doi: 10.1371/journal.pone.0166128 (PMC5113044; doi:10.1371/journal.pone.0166128)
Supplement: S1 Fig — (A) Effects of inhibitors on OsmC esterase activities using 4-nitrophenol benzoate (1.5 mM) as substrate. (B) Effects of metal salts on OsmC esterase activities using 4-nitrophenol benzoate (1.5 mM) as substrate. ΔEstCAL-Blue, ΔEstCA-black, ΔEstRD-green, ΔEstO-grey, ΔEstEM-orange, ΔEstRM-red, ΔEstLA-magenta. Results were plotted as percentages of activity relative to measured activity when no inhibitor or metals salts were present. Results are presented as means ± S.D. of triplicate experiments. (PDF) [file pone.0166128.s001.pdf]

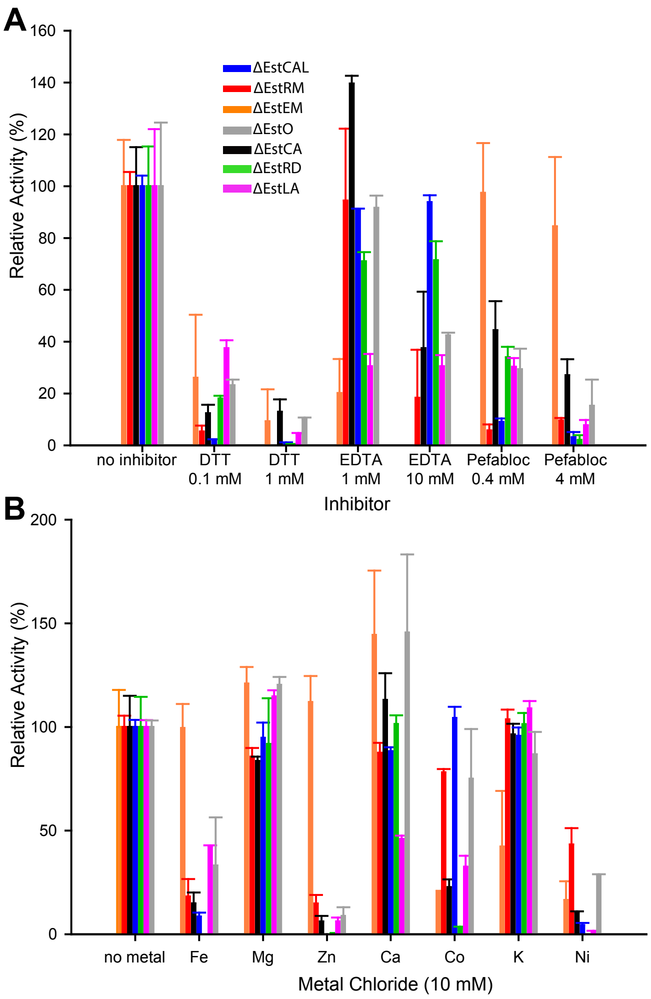

S1 figure. Relative activity of ester hydrolysis after incubation with known inhibitors and metal salts on activity of truncated OsmC esterases.
