# Supplementary figures and images for: Characterisation of a New Family of Carboxyl Esterases with an OsmC Domain
Source: PLoS One. 2016 Nov 16;11(11):e0166128. doi: 10.1371/journal.pone.0166128 (PMC5113044; doi:10.1371/journal.pone.0166128)

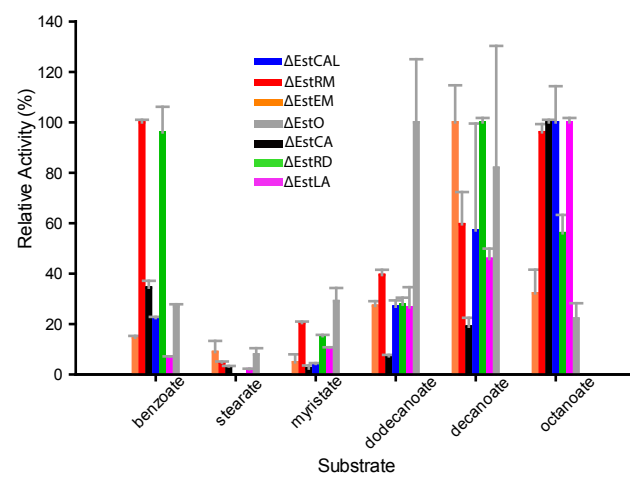

**S2 figure. Relative substrate specificities of OsmC esterases.**

Supplement: S2 Fig — Enzyme assays were performed with 4-NP esters of varying chain length (C8-C18) and an aromatic ester (benzoate) as substrates (1.5 mM). ΔEstCAL-Blue, ΔEstCA-black, ΔEstRD-green, ΔEstO-grey, ΔEstEM-orange, ΔEstRM-red, ΔEstLAmagenta. Results were plotted as percentages of activity relative to substrate with highest measured activity of individual esterases. Results are presented as means ± S.D. of triplicate experiments. (PDF) [file pone.0166128.s002.pdf]

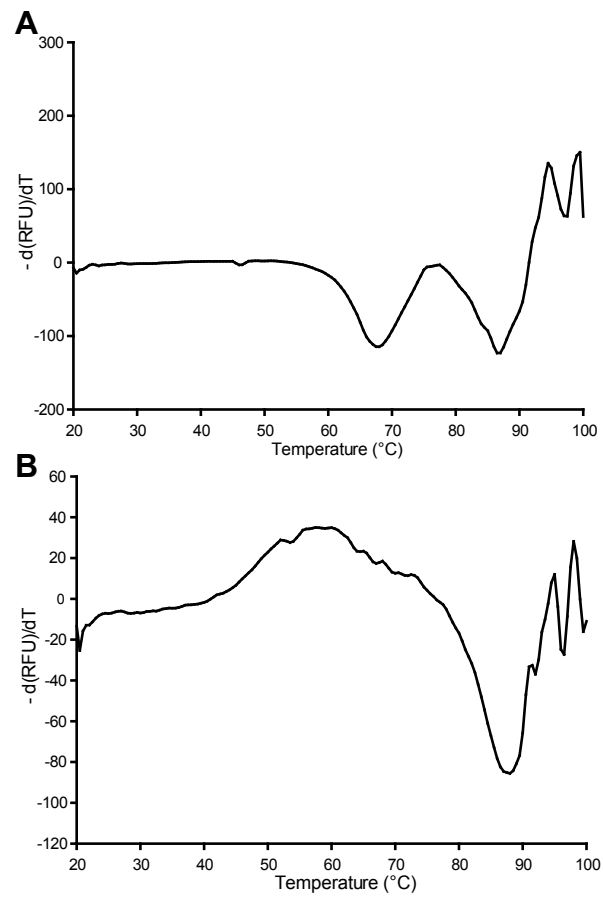

**S3 figure. Thermal stability of full length and  $\Delta$ EstRM proteins.**

Supplement: S3 Fig — Thermal melting profiles for (A) full length and (B) ΔEstRM proteins. Unfolding of esterases was monitored between 20 and 100°C using SYPRO Orange fluorescent dye. The gradients of esterase unfolding were plotted as a function of temperature. Results are presented as means of triplicate experiments. (PDF) [file pone.0166128.s003.pdf]

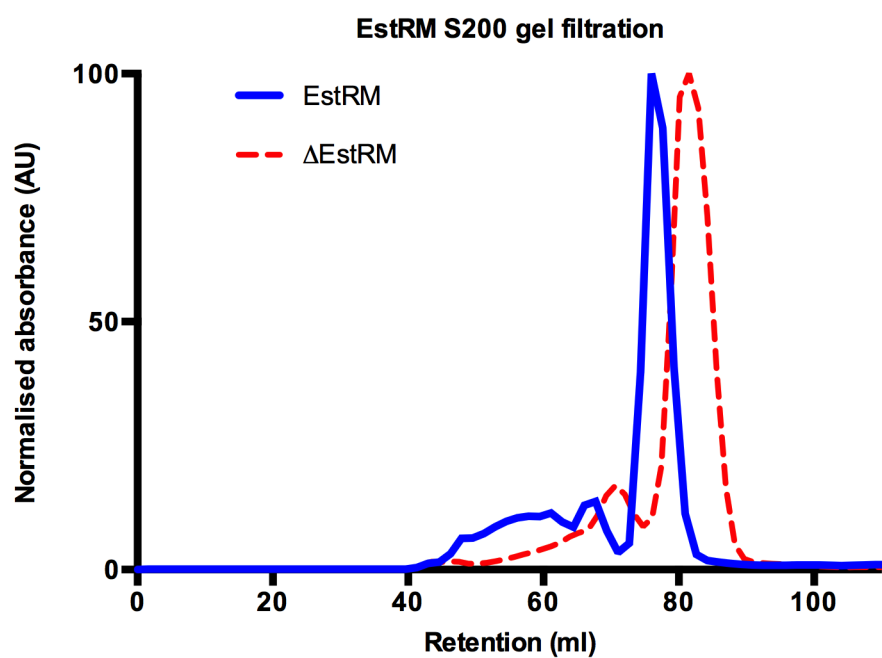

**S6 figure. S200 size-exclusion gel-filtration chromatography of EstRM and  $\Delta$ EstRM.**

Supplement: S6 Fig — Relative absorbance at 280nm is plotted against elution volume for both the full length EstRM and the ΔEstRM truncation. The major peaks at 76 ml (EstRM) and 82 ml (ΔEstRM) are consistent with the monomer size, while the minor peaks at 68 (EstRM) and 72 ml (ΔEstRM) represent a minor polulation of dimeric protein. The full-length EstRM trace shows a proportion of the protein aggregating into higher-order oligomers. (PDF) [file pone.0166128.s006.pdf]
