# Supplementary material for: Characterisation of a New Family of Carboxyl Esterases with an OsmC Domain
Source: PLoS One. 2016 Nov 16;11(11):e0166128. doi: 10.1371/journal.pone.0166128 (PMC5113044; doi:10.1371/journal.pone.0166128)
Supplement: S4 Fig — (A) Enzymes were pre-incubated with 10 mM of EDTA, DTT, Pefabloc or PMSF for 1 hour before addition to substrate in assay buffer. (B) Enzymes were pre-incubated with 10 mM of CuI, CuII, Fe, Ni, Zn, Co, Mg, K, Ca, or Na for 1 hour before addition to substrate in assay buffer. Substrate used to monitor activity was 4-nitrophenyl-octanoate (1 mM). Full-length EstRM enzyme is shown in black and truncated EstRM (ΔEstRM) in grey. Results are presented as means ± S.D. of triplicate experiments. (PDF) [file pone.0166128.s004.pdf]

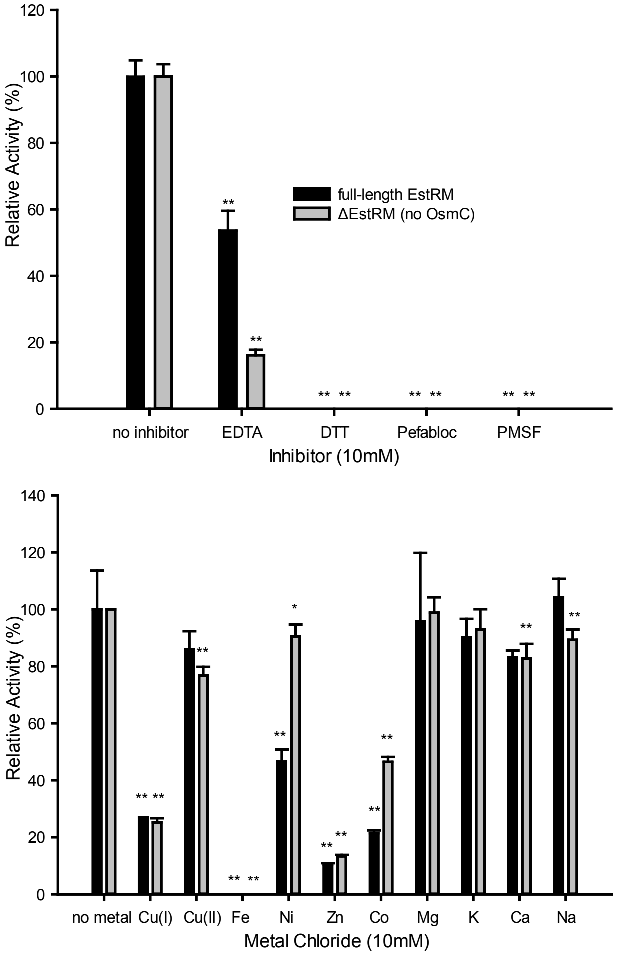

S4 figure. Relative activity of ester hydrolysis after incubation with 10 mM known inhibitors and metal salts on activity of full length and truncated EstRM.
