# Supplementary material for: Characterisation of a New Family of Carboxyl Esterases with an OsmC Domain
Source: PLoS One. 2016 Nov 16;11(11):e0166128. doi: 10.1371/journal.pone.0166128 (PMC5113044; doi:10.1371/journal.pone.0166128)
Supplement: S5 Fig — The protein backbone is shown as a cartoon view with the cysteine residues shown as sticks. The 2mFo-DFc electron density for this region is shown as an orange mesh at 1.5 σ. It is clear from the electron density that this disulphide is only partially occupied in this structure as multiple conformations of the sulphur atom are visible. (PDF) [file pone.0166128.s005.pdf]

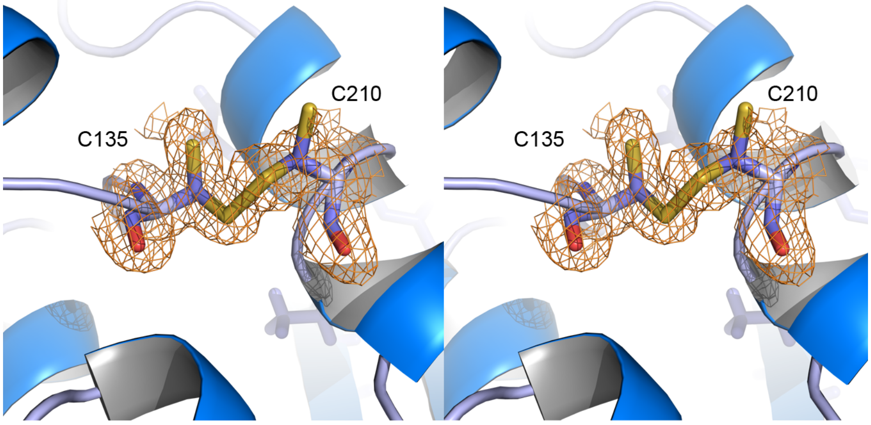

S5 figure. Wall-eyed stereo view of the intramolecular disulphide present in the ΔEstRM crystal structure.
